# Giant cardiac intracavitary mass and suspect rhabdomyomatosis in newborn, in developing country

**DOI:** 10.11604/pamj.2021.40.22.30054

**Published:** 2021-09-08

**Authors:** Federica Iezzi, James Munene

**Affiliations:** 1 Department of Paediatric and Congenital Cardiac Surgery and Cardiology, Azienda Ospedaliero Universitaria, Ospedali Riuniti Ancona (Umberto I, GM Lancisi, G Salesi), Ancona, Italy,; 2 Department of Cardiothoracic and Vascular Surgery, Kenyatta National Hospital and University of Nairobi, Nairobi, Kenya

**Keywords:** Rhabdomyoma, newborn, bradycardia

## Image in medicine

After a prenatal diagnosis of left ventricle apex giant mass, a late preterm (38 weeks) baby with a birth weight of 2980 grams, developed refractory bradycardia immediately after birth. After birth a 12 lead electrocardiogram (ECG) revealed sinus bradycardia. Peaked P waves. The duration of particle quantification (PQ) interval was 110 m/sec. The following Q wave, R wave and S wave (QRS) complex was normal, with ST-elevation in inferior leads. During continuous monitoring ECG a symptomatic bradycardia has been documented with phases of respiratory sinus arrhythmia with heart rate about 60-70 bpm. Echocardiography showed a large, non-capsulated 16x21 mm mass, with a large and thin base of implant and ovoid morphology occupying most of the left ventricle cavity. Clinical presentation resembled that of critical aortic stenosis. For persistent and symptomatic bradycardia, not responsive to medical treatment, and for initial signs of heart failure, the baby underwent urgent complete surgical resection of the intracardiac mass, during the second day of life. The ascending aorta was transected. Through the aortic valve, the left ventricular outflow tract was explored. A large homogeneous white mass was found just below the leaflets of the aortic valve. The large tumor infiltrated the ventricular septum and protruded into the left ventricular outflow tract, occluding 90% of its diameter. The cardiac mass was detached from the ventricular septum and the protruding portion was completely resected. Histological examination of the mass confirmed the diagnosis of rhabdomyoma.

**Figure 1:**
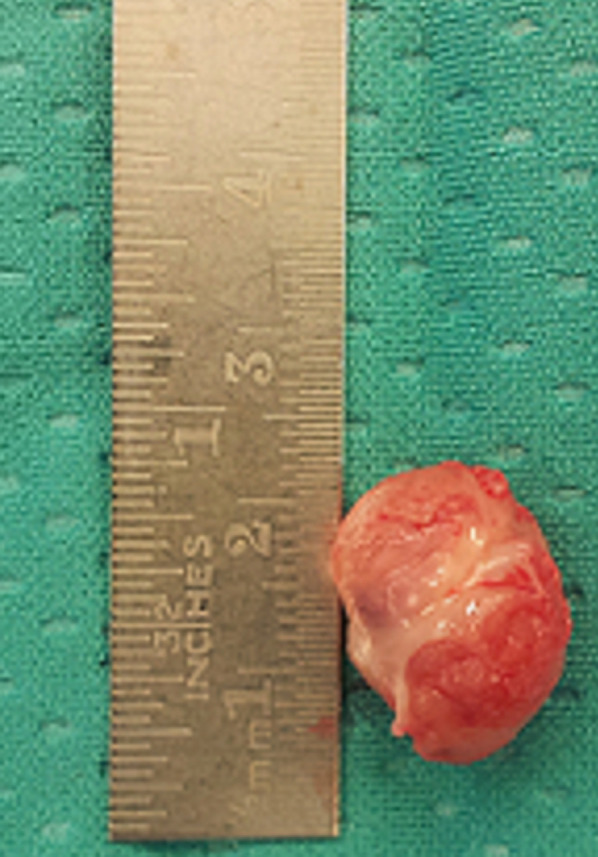
surgically resected cardiac rhabdomyoma

